# Future Perspectives on Radiomics in Acute Liver Injury and Liver Trauma

**DOI:** 10.3390/jpm14060572

**Published:** 2024-05-27

**Authors:** Maria Chiara Brunese, Pasquale Avella, Micaela Cappuccio, Salvatore Spiezia, Giulia Pacella, Paolo Bianco, Sara Greco, Luigi Ricciardelli, Nicola Maria Lucarelli, Corrado Caiazzo, Gianfranco Vallone

**Affiliations:** 1 Department of Medicine and Health Science “V. Tiberio”, University of Molise, 86100 Campobasso, Italy; mariachiarabrunese@gmail.com (M.C.B.);; 2 Department of Clinical Medicine and Surgery, University of Naples Federico II, 80131 Naples, Italy; 3 Hepatobiliary and Pancreatic Surgery Unit, Pineta Grande Hospital, 81030 Castel Volturno, Italy; 4 Interdisciplinary Department of Medicine, Section of Radiology and Radiation Oncology, University of Bari “Aldo Moro”, 70124 Bari, Italy; 5 AORN dei Colli, 80131 Naples, Italy

**Keywords:** artificial intelligence, liver trauma, acute liver injury, drug-induced liver injury, radiomics, abdominal trauma

## Abstract

**Background**: Acute liver injury occurs most frequently due to trauma, but it can also occur because of sepsis or drug-induced injury. This review aims to analyze artificial intelligence (AI)’s ability to detect and quantify liver injured areas in adults and pediatric patients. **Methods**: A literature analysis was performed on the PubMed Dataset. We selected original articles published from 2018 to 2023 and cohorts with ≥10 adults or pediatric patients. **Results**: Six studies counting 564 patients were collected, including 170 (30%) children and 394 adults. Four (66%) articles reported AI application after liver trauma, one (17%) after sepsis, and one (17%) due to chemotherapy. In five (83%) studies, Computed Tomography was performed, while in one (17%), FAST-UltraSound was performed. The studies reported a high diagnostic performance; in particular, three studies reported a specificity rate > 80%. **Conclusions**: Radiomics models seem reliable and applicable to clinical practice in patients affected by acute liver injury. Further studies are required to achieve larger validation cohorts.

## 1. Introduction

Acute liver injury is defined as severe acute necrosis of hepatic cells, mostly due to liver trauma or acute liver failure, but it is also one of the most common complications of sepsis [1,2,3]. Approximately 10% of abdominal trauma involves liver parenchyma [4,5,6]. Integrating clinical and biochemical parameters is mandatory to achieve an accurate diagnosis and to quantify the parenchymal damage [7,8].

Focused Assessment with Sonography in Trauma UltraSound (FAST-US) is the first-level exam for traumatized patients, and, whether associated with chest X-ray or pelvis X-ray, FAST-US has a sensitivity and specificity of 90% and 93%, respectively, to identify major injuries in patients who experienced trauma [9,10,11]. Although the latest evidence about FAST-US is comforting, the choice of avoiding Contrast-Enhanced Computed Tomography (CE-CT) as a screening exam in an emergency is still controversial [12,13,14].

At the same time, to better organize the decision making for a traumatized patient, the pre-hospital phase should employ triage tools [15,16,17,18].

Nowadays, in the emergency context, CT is still considered the gold standard of diagnosis to evaluate and quantify major and minor liver injuries and any associated organ ruptures or intra-abdominal bleeding [19,20]. CE-CT allows radiologists to assess the percentage of parenchymal injuries and then estimate the degree of damage and plan a surgical or medical approach [21]. Surely, if available, hybrid operating rooms also best benefit the fast execution of CE-CT of the interventional and surgical procedures [22,23,24,25,26]. CE-CT is also useful for monitoring injured patients after treatment to evaluate infective or hemorrhagic complications [27].

**Figure 1:**
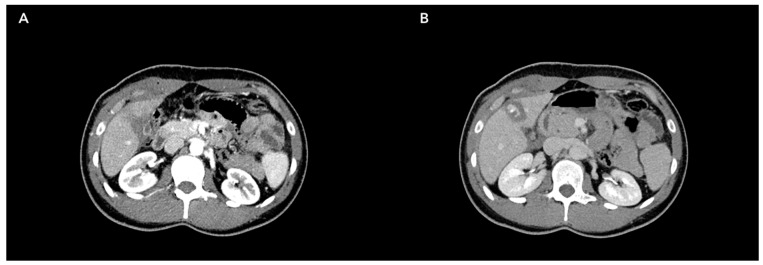
Liver trauma due to a penetrating body;: bleeding involved IVb and V hepatic segments. (**A**) Arterial phase. (**B**) Portal phase.

**Figure 2:**
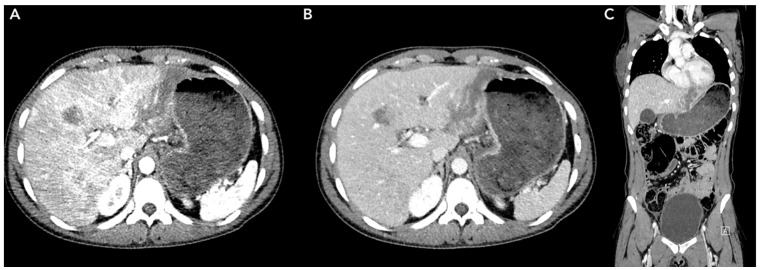
Liver trauma due to a car accident; a grade V injury based on the AAST score has been detected at the II and III liver segments. (**A**) Arterial phase. (**B**,**C**) Portal phase.

**Figure 3:**
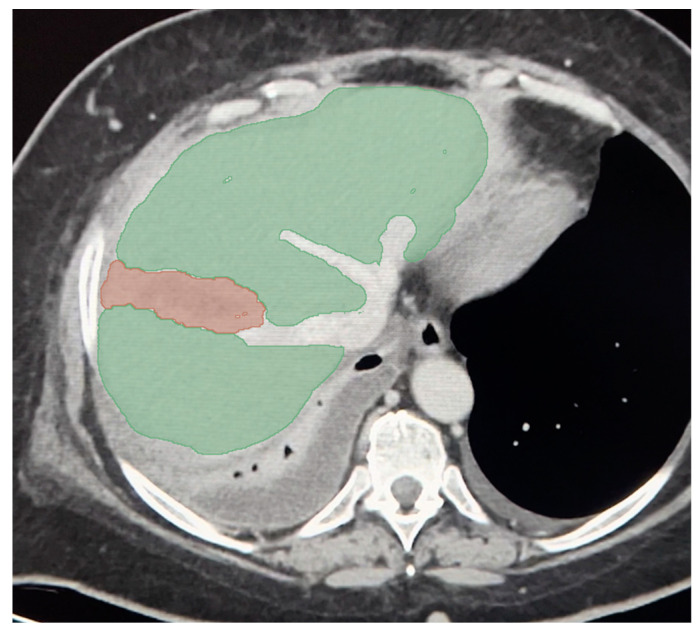
Manual segmentation of injured area (red) and liver parenchyma (green) excluding vessels.

The American Association for the Surgery of Trauma (AAST) injury scoring scales for liver injury are based on CE-CT, and they are the primary tool used in clinical practice [28] (Figure 1, Figure 2 and Figure 3). The scale includes six grades representing the severity of injury: grade I subcapsular hematoma (<10% of surface area and >1 cm of depth); grade II (10–50% of surface area, intrahepatic hematoma of 10 cm of axial maximum diameter, 1–3 cm depth); grade III (>50% of surface area, >10 cm axial maximum diameter, >3 cm of depth), grade IV (25–75% lobe disruption, active bleeding), and grade V (>75% lobe disruption, cava, and suprahepatic vein rupture; grade VI liver avulsion) [5,29,30,31]. The AAST grading system is also the grading score chosen for the pediatric population [32,33,34].

International guidelines have evaluated the therapeutic approach for each grade, even if the decision-making process remains strictly related to hemodynamic stability and the availability of vascular radiologists [35,36,37].

However, in the emergency scenario, acute liver injury may also not be due to trauma. One of the most common presentations of acute liver injury is related to septic patients [38], who are supposed to have a mortality rate of approximately 2–40% [39]. Nowadays, the evaluation of liver function in septic patients is based on serological markers, but their correlation with CT imaging improves the diagnostic power [38,39]. CT is mandatory to quantify the parenchymal damaged volume, the capsule, and the blood vessels’ integrity.

Nowadays, estimating the injured parenchymal volume requires manual segmentation of the region of interest (ROI), as for the evaluation of the oncological disease [40,41,42,43,44,45]. Manual ROI defining requires a long work time, and a dedicated operator is often unavailable in small regions’ Hub centers and peripheral centers in an emergency context [45,46,47].

Artificial intelligence (AI) tools have already been developed in many fields of medicine to manage different kinds of clinical data [48,49,50,51,52]. In the era of AI, radiomics has been proposed as a promising non-invasive tool able to translate images into data from radiological features to radiomics features, which can be analyzed using dedicated models [53]. Radiomic features can be extracted from different imaging methodologies, including US, CT, or MRI [53,54,55,56,57,58,59,60]. The models are built on standard steps: image upload, ROI definition, feature extraction, feature selection, model building, and testing.

The published literature on acute abdominal injury shows that the prognostic factors are not yet clear, which influences the outcome for patients treated in an emergency context [61,62].

Several studies have already investigated the role of AI in the emergency context to achieve a more accurate diagnosis, a differential diagnosis, or a prognosis or to choose personalized therapeutic strategies for predicting treatment outcomes [63,64].

This review aims to analyze the AI tools dedicated to acute liver injury to explore their ability to detect and quantify the injured areas and attribute the right AAST score in adults and pediatric patients in a short time and with suitable accuracy.

## 2. Methods

We performed a literature research on the PubMed Dataset (US National Library of Medicine, http://www.ncbi.nlm.nih.gov/PubMed (accessed on 10 January 2024)), using the subsequent keywords:

(((artificial intelligence) OR (radiomics) OR (neural networks) OR (machine learning) OR (deep learning) OR (texture analysis)) AND ((liver OR hepatic) AND (((injury) OR (rupture) OR (damage) AND (related OR induced)) OR (trauma))) AND (“English”[Language]).

The publication date was set from 2018 to September 2023. Original articles were included, while editorials, letters, reviews, and case reports were excluded. It was also decided to include case series with a minimum of 10 patients in the study. Articles were first included based on the title and abstract, and then a full-text read was performed. All patients signed an informed consent allowing for the anonymous scientific use of clinical data and images reported in our study.

## 3. Results

At the end of the literature research, we found 367 studies. Two-hundred and seventy-six studies were evaluated as pertinent from 2018 to September 2023. After title and abstract screening, we included 10 studies. At the end of the full-text examination, we included only 6 papers that address the application of radiomics tools on diagnostic imaging concerning the diagnosis, the prognosis, and the treatment of acute liver injury [64,65,66,67,68,69] (Table 1).

The included studies analyze the automated detection, segmentation, and quantification of liver-injured areas in both adult and pediatric populations and the automatic detection of major artery injuries.

### 3.1. Automated Diagnosis of Liver Trauma

CT is considered the gold-standard technique to determine AAST grade by evaluating the fraction of liver parenchyma injured [70]. The study conducted by Farzaneh et al. [64] completely addresses the diagnosis, automated segmentation, and quantification of damage. In the study, 77 patients were included with an average patient age of 41 years. Among 77 patients, 34 patients had liver injury, and 43 did not have evidence of liver damage.

Even if the standard CT exam provides both an arterial and portal phase, in that study, the model was built only on the portal phase. The study was divided into liver segmentation and injury segmentation. As a ground truth, the damaged area and the entire parenchyma were manually segmented.

The aim was to automatically estimate the percentage of the volume of injured parenchyma by fractionating this with the whole parenchymal volume. Both of the volume measurements were performed through automatic segmentation. The proposed methodology achieved the best recall on liver injury > 5% of 74%, while, for the entire sample, the recall was 53%. However, the model correctly classified the 90% of healthy patients [64].

Concerning the pediatric population, S. Huang et al. performed a study on 170 children affected by blunt liver trauma. Also, the study focused on the automated quantitative assessment of liver damage and parenchyma segmentation [65].

The model was built as in the precedent study: only portal-phase images and ground-truth labels were obtained by two radiologists who segmented the entire parenchyma and the damaged areas. Most liver lesions were classified as AAST II and III. The diagnostic performance of liver trauma volume was recall of 93.1% and specificity of 91.3%. Both studies [64,65] achieved a great specificity; therefore, the probability of overdiagnosis of liver trauma is minimal. Both models [64,65] have the strong limitation of being built on a retrospective cohort, so their promising results need to be tested in clinical practice on a prospective cohort. In both studies [64,65], which automatized the segmentation with great accuracy, the radiologists could check that the model works and focus their attention on the AAST score.

Concerning the application of radiomics to interventional radiology and treatment strategy, Dreizin et al. proposed a methodology to predict the major hepatic arterial injury in a cohort of patients who underwent angiography following a primary CT scan [66].

The patient dataset was composed of 73 stable adult patients who had undergone CT and then angiography; therefore, unstable patients who underwent laparotomy or angiography upfront were excluded. In total, 41 patients had been diagnosed with arterial injury and 40 underwent angiography with arterial embolization. The logistic regression showed that contrast extravasation and the index of liver damage are significant and independent predictors of major hepatic arterial injury. Once the automatic segmentation of liver damage was performed, the deep-learning model was built. The sensitivity achieved was 83% and the specificity was 84%, with a global accuracy rate of 84%. The study [66] demonstrated that precise personalized decisions could improve the diagnostic performance of standard protocols, thus improving the missed diagnosis of injury and delayed surgical or interventional approaches.

In the large field of radiomics, ultrasomics has already been explored in the evaluation and differential diagnosis of oncological patients, and it has also been applied to FAST-US exams [67]. Levy et al. [67] proposed a study about the qualitative and quantitative assessment of abdominal trauma in unstable patients who would not undergo CT scans [67]. As known, FAST-US evaluates the presence of fluid in the right upper quadrant and the left upper quadrant and in pelvis [9,67].

In the study, the authors focused on the hepatorenal and splenorenal space to evaluate the presence or absence of fluid through the convolutional neural network. In total, 109 patients were retrospectively reviewed, and 6608 images were analyzed. The results achieved on the validation cohort were 95% accuracy, 94% sensitivity, and 100% specificity in the diagnosis of fluid presence [67].

### 3.2. Sepsis-Induced Liver Injury

Beyond trauma, in an emergency context, sepsis can commonly cause acute liver injury in about 34% of patients affected due to inadequate liver perfusion [71,72,73].

However, few studies are treating the early diagnosis of sepsis-induced liver injury, and the quantitative evaluation of liver damage is still limited to serological markers (bilirubin concentration > 2 mg/dL and coagulation INR > 1.5) [74].

Of course, exactly as for accidental liver injuries, as the main cause of liver injury after sepsis is insufficient liver perfusion, the contrast-enhanced CT scan is the gold standard for the diagnosis and quantification of parenchymal damage [68].

Wang et al. [68] proposed a study to evaluate the automatic segmentation and to improve the identification and quantification of liver injury in patients affected by sepsis. In the study, 92 patients were enrolled, including 50 (54%) patients with non-acute liver injury and 42 (46%) patients with acute liver injury. FCN (full convolutional neural network) improved the accuracy of the segmentation of traditional convolutional neural networks, achieving precision and recall rates of 91% and 88%, respectively [68].

### 3.3. Drug-Induced Liver Injury

Drug-induced liver injury (DILI) is an uncommon cause of acute liver failure that is very challenging to detect and treat [75].

The diagnosis of DILI is mostly based on clinical and serological markers; however, several studies have proposed Machine Learning methods to improve the diagnostic performance of radiological methodologies to detect DILI early and to differentiate it from chronic liver diseases [76,77]. Concerning the application of AI on radiological images, to our knowledge, there are not many CT–radiomics applications on cisplatin or, generally, chemotherapy-induced liver injury. Alessandrino et al. [69] conducted a study on CT images to evaluate the prediction of 5-Fluorouracil-induced liver toxicity through texture analysis.

The results showed different features, such as the mean, entropy, skewness, and SD, significantly related to the early diagnosis of liver injury. The study is an example of the ability of radiomics to analyze liver parenchyma on CT images [77].

Other applications of AI to DILI concern antiviral drugs, antibiotics, or nonsteroidal anti-inflammatory drugs (NSAIDs), but they are not based on radiological images yet [78,79,80,81].

## 4. Discussion

Contrast-enhanced CT (CE-CT) plays a significant role in the management of liver trauma, as it is mandatory to confirm the diagnosis, to localize the injured segment, and to exclude any other organ injuries or peritoneal bleeding [82,83].

The 3D reconstructions ensure a reliable quantification of the volume injured and support the classification of the damage using the AAST scale [84]. A different assessment of the severity grade impacts the management and the decision of whether a conservative, interventional, or surgical treatment is required [29,84,85].

A potential negative outcome from a conservative approach may derive from a disagreement between the CT scan and the intraoperative findings [86,87]. However, a CT scan is also needed to evaluate the response to treatment and to monitor the progression or resolution of the liver injuries during follow-up [88,89]. Therefore, ensuring the highest sensitivity is mandatory to avoid misdiagnosis and for treatment planning [90,91].

The included studies in our review have three main results.

First, the model proposed by Huang et al. [65] demonstrates a good liver trauma segmentation performance, with an overall sensitivity of 79.5%. The performance proportionally increases among the AAST grades. In particular, the lowest recall (sensitivity) belongs to grade I (50%), while the highest recall (sensitivity) belongs to grade V (85%). This difference is also due to the small number of patients enrolled with a grade I injury. It can be speculated that the achieved accuracy is comparable to the one of the radiologists or clinicians, but underlying liver disease, such as liver steatosis or cirrhosis, may significantly impact the attenuation of liver parenchyma in CE-CT.

Second, all of the included studies performed an automatic segmentation of the entire liver parenchyma to measure the volume injured correctly. Automatic segmentation gives the possibility to clinicians to obtain an answer from the model in the shortest time. The segmentation was also built to perform a 3D quantitative analysis [64,65,66]. Given this, the models are ready for external validation in a real prospective cohort.

Third, the feasibility of one model to detect the injured lesion is verified even in trauma patients and in non-trauma patients, because it can assess liver injury and its severity quickly [65].

A preoperative CT scan is also needed in cases of high-grade injuries to evaluate the future liver remnant after resection to personalize the treatment strategy [92,93]. It is also important to underline that in general surgical procedures in an emergency context, an inefficient diagnosis may require an urgent laparoscopy [94,95].

Moreover, during the monitoring, the radiologists’ experience must also concern persistent bleeding, haemobilia, and biliary fistula [96,97,98,99,100,101]. Consequently, these complications must be treated to avoid cole-peritoneum and sepsis, which can also worsen the volume injured [97,98,99,100,101,102,103,104,105].

Considering that the most common site of injury is the right liver and that the complications may require significant experience in hepato-biliary (HB) surgery, the first diagnostic exam can also be mandatory to transfer stable patients to a Liver Unit to ensure the best treatment [106,107,108].

Furthermore, especially in post-depacking bleeding, the surgical approach may consist of major hepatic resection and portal vein ligation if the interventional approach did not work [109,110,111,112,113].

This surgical technique requires an important learning curve because it is a prerogative of HB surgeons trained in oncological resections or liver transplantations [114,115,116,117,118,119]. To reduce healthcare migration and enhance the patient survival rate, it could organize the Hub&Spoke learning program, similarly to other general surgery procedures [120,121,122,123,124,125,126,127,128,129]. However, in the era of minimally invasive surgery [130,131], referral centres should also employ robotic or laparoscopic approaches in liver trauma [132,133,134,135,136,137,138,139,140,141]. In order to create a personalized treatment for different patients, a study treated liver trauma affecting the pediatric population [65].

However, to our knowledge, there are not already available radiomics models to ensure personalized treatment strategies in an emergency context in the elderly population. As known, geriatric surgery is based on different therapeutic algorithms from general surgery, and the huge variability among this population is strictly related to the different performance and ASA scores [142,143,144,145,146,147,148].

In the literature, no studies are reported on radiomics applied to the management of or response to conservative, interventional, or surgical therapies. For this reason, future further analyses are also required that consider the different surgical approaches available [149,150,151].

Concerning the application of radiomics in sepsis-induced liver injury, it is surely more challenging, because it also requires a differential diagnosis between liver lesions, often in oncological patients. Patients who develop acute liver injury are more likely to have a history of drinking, as chronic alcohol consumption causes liver steatohepatitis and then cirrhosis and consequent liver dysfunction [152]. At the moment of developing a septic state, the ability of the liver to compensate for the stress is reduced in patients with an underlying chronic liver disease [153]. Therefore, ulterior damage caused by inefficient perfusion is not well-tolerated [154].

The diagnostic power of CT is also reduced by the attenuation of liver parenchyma at CT due to steatosis or cirrhosis, so the diagnosis is more challenging [155,156]. As a delayed diagnosis can be significant for patients’ survival, a radiomic model is one of the solutions to the problem [157,158,159,160].

Furthermore, a delayed diagnosis can cause delayed treatment and more severe damage, and, consequently, a longer stay in the Intensive Care Unit (ICU) [161,162,163].

## 5. Conclusions

The radiomics model proposed in both liver trauma and sepsis-related liver injury has the great value of being built on automatic segmentation. Their methodology is reliable and applicable in clinical practice, but prospective multicenter validation is still required.

## Figures and Tables

**Table 1:** Artificial intelligence applications to acute liver injury after trauma, sepsis, and drug-induced liver injury.

Author(s)	Year	Population	No. of Patients	Liver Injury Types	Imaging	Aims	Results
Farzaneh et al. [64]	2022	Adults	77	- 34 liver trauma - 43 no liver damage	CT	- Injured parenchyma volume - Parenchyma segmentation	- Recall 74%
Huang et al. [65]	2022	Children	170	- 170 blunt liver trauma	CT	- Injured parenchyma volume - Parenchyma segmentation	- Recall 93% - Specificity 91%
Dreizin et al. [66]	2021	Adults	73	- 170 blunt liver trauma	CT	- Arterial injury diagnosis - Parenchyma segmentation	- Sensitivity 83% - Specificity 84% - Global accuracy 84%
Levy et al. [67]	2023	Adults	109	- 109 abdominal trauma	FAST-US	- Presence/absence of abdominal fluid	- Sensitivity 94% - Specificity 100% - Global accuracy 95%
Wang et al. [68]	2022	Adults	92	- 50 patients with non-acute liver injury during sepsis - 42 acute liver injury	CT	- Parenchyma segmentation	- Recall 91% - Precision 88%
Alessandrino et al. [69]	2019	Adults	43	- 43 5FU administration	CT	- Parenchyma segmentation	NA

**Abbreviations**: 5FU, 5-Fluorouracil; CT, Computed Tomography; NA, not available.

## Data Availability

Not applicable.

## References

[1] Koch D.G., Speiser J.L., Durkalski V., Fontana R.J., Davern T., McGuire B., Stravitz R.T., Larson A.M., Liou I., Fix O. (2017). The Natural History of Severe Acute Liver Injury. Am. J. Gastroenterol..

[2] Renzi F., Reitano E., Franca D., Chiara O., Cimbanassi S. (2022). Trauma, alcohol and drugs misuse in car and motorcycle drivers: A prevalence study in a level one trauma center. Updates Surg..

[3] Russo M.W., Galanko J.A., Shrestha R., Fried M.W., Watkins P. (2004). Liver transplantation for acute liver failure from drug induced liver injury in the United States. Liver Transplant..

[4] Reitano E., Bini R., Difino M., Chiara O., Cimbanassi S. (2022). Nine year in-hospital mortality trends in a high-flow level one trauma center in Italy. Updates Surg..

[5] Coccolini F., Coimbra R., Ordonez C., Kluger Y., Vega F., Moore E.E., Biffl W., Peitzman A., Horer T., Abu-Zidan F.M. (2020). Liver trauma: WSES 2020 guidelines. World J. Emerg. Surg..

[6] Özpek A., Yıldırak M.K., Ezberci F. (2024). Hollow viscus injury due to blunt abdominal trauma: A tertiary trauma center experience. Ulus. Travma Acil Cerrahi Derg..

[7] McGill M.R., Jaeschke H. (2019). Biomarkers of drug-induced liver injury. Adv. Pharmacol..

[8] Senior J.R. (2012). Alanine aminotransferase: A clinical and regulatory tool for detecting liver injury-past, present, and future. Clin. Pharmacol. Ther..

[9] Reitano E., Granieri S., Sammartano F., Cimbanassi S., Galati M., Gupta S., Vanzulli A., Chiara O. (2022). Avoiding immediate whole-body trauma CT: A prospective observational study in stable trauma patients. Updates Surg..

[10] van Vugt R., Kool D.R., Deunk J., Edwards M.J. (2012). Effects on mortality, treatment, and time management as a result of routine use of total body computed tomography in blunt high-energy trauma patients. J. Trauma Acute Care Surg..

[11] Jiang L., Ma Y., Jiang S., Ye L., Zheng Z., Xu Y., Zhang M. (2014). Comparison of whole-body computed tomography vs selective radiological imaging on outcomes in major trauma patients: A meta-analysis. Scand. J. Trauma Resusc. Emerg. Med..

[12] Rubin G.D. (2014). Computed tomography: Revolutionizing the practice of medicine for 40 years. Radiology.

[13] Rocca A., Komici K., Brunese M.C., Pacella G., Avella P., Di Benedetto C., Caiazzo C., Zappia M., Brunese L., Vallone G. (2024). Quantitative ultrasound (QUS) in the evaluation of liver steatosis: Data reliability in different respiratory phases and body positions. Radiol. Med..

[14] Caputo N.D., Stahmer C., Lim G., Shah K. (2014). Whole-body computed tomographic scanning leads to better survival as opposed to selective scanning in trauma patients: A systematic review and meta-analysis. J. Trauma Acute Care Surg..

[15] Bagnato C., Ranzato K., Giarraca A., Restelli P., Saronni S., Gadda G., Chiara O., Cimbanassi S. (2022). A prospective study comparing two methods of pre-hospital triage for trauma. Updates Surg..

[16] James M.K., Clarke L.A., Simpson R.M., Noto A.J., Sclair J.R., Doughlin G.K., Lee S.W. (2019). Accuracy of pre-hospital trauma notification calls. Am. J. Emerg. Med..

[17] Lampi M., Junker J., Berggren P., Jonson C.O., Vikström T. (2017). Pre-hospital triage performance after standardized trauma courses. Scand. J. Trauma Resusc. Emerg. Med..

[18] Segalini E., Morello A., Leati G., Di Saverio S., Aseni P. (2022). Primary angioembolization in liver trauma: Major hepatic necrosis as a severe complication of a minimally invasive treatment—A narrative review. Updates Surg..

[19] Rouy M., Julien C., Hamouda I., Massalou D., Bege T., Leone M., Berdah S., Barbois S., Girard E., Arvieux C. (2022). Predictive factors of non-operative management failure in 494 blunt liver injuries: A multicenter retrospective study. Updates Surg..

[20] Cheng C.T., Lin H.H., Hsu C.P., Chen H.W., Huang J.F., Hsieh C.H., Fu C.Y., Chung I.F., Liao C.H. (2024). Deep Learning for Automated Detection and Localization of Traumatic Abdominal Solid Organ Injuries on CT Scans. J. Imaging Inf. Med..

[21] Parenteau C.S., Ehrlich P., Ma L., Su G.L., Holcombe S., Wang S.C. (2013). The quantification of liver anatomical changes and assessment of occupant liver injury patterns. SAE Tech. Pap..

[22] Kirkpatrick A.W., Vis C., Dubé M., Biesbroek S., Ball C.G., Laberge J., Shultz J., Rea K., Sadler D., Holcomb J.B. (2014). The evolution of a purpose designed hybrid trauma operating room from the trauma service perspective: The RAPTOR (Resuscitation with Angiography Percutaneous Treatments and Operative Resuscitations). Injury.

[23] Loftus T.J., Croft C.A., Rosenthal M.D., Mohr A.M., Efron P.A., Moore F.A., Upchurch G.R., Smith R.S. (2021). Clinical Impact of a Dedicated Trauma Hybrid Operating Room. J. Am. Coll. Surg..

[24] Jin H., Lu L., Liu J., Cui M. (2022). A systematic review on the application of the hybrid operating room in surgery: Experiences and challenges. Updates Surg..

[25] Balch J.A., Loftus T.J., Ruppert M.M., Rosenthal M.D., Mohr A.M., Efron P.A., Upchurch G.R., Smith R.S. (2023). Retrospective value assessment of a dedicated, trauma hybrid operating room. J. Trauma Acute Care Surg..

[26] Prichayudh S., Rajruangrabin J., Sriussadaporn S., Pak-Art R., Kritayakirana K., Samorn P., Narueponjirakul N., Uthaipaisanwong A., Aimsupanimitr P., Chaisiriprasert P. (2023). Trauma Hybrid Operating Room (THOR) shortened procedure time in abdominopelvic trauma patients requiring surgery and interventional radiology procedures. Injury.

[27] Machado Lessa C.L., Branchini G., Moreira Delfino I., Ramos Voos M.H., Teixeira C., Hoher J.A., Nunes F.B. (2024). Comparison of Sepsis-1, 2 and 3 for Predicting Mortality in Septic Patients of a Middle-Income Country: A Retrospective Observational Cohort Study. J. Intensive Care Med..

[28] Croce M.A., Fabian T.C., Kudsk K.A., Baum S.L., Payne L.W., Mangiante E.C., Britt L.G. (1991). AAST organ injury scale: Correlation of CT-graded liver injuries and operative findings. J. Trauma.

[29] Rocca A., Andolfi E., Zamboli A.G.I., Surfaro G., Tafuri D., Costa G., Frezza B., Scricciolo M., Amato M., Bianco P. (2019). Management of Complications of First Instance of Hepatic Trauma in a Liver Surgery Unit: Portal Vein Ligation as a Conservative Therapeutic Strategy. Open Med..

[30] Roberts R., Sheth R.A. (2021). Hepatic trauma. Ann. Transl. Med..

[31] Campbell B.R., Rooney A.S., Krzyzaniak A., Lee J.J., Carroll A.N., Calvo R.Y., Peck K.A., Martin M.J., Bansal V., Sise M.J. (2024). To the point: Utility of laparoscopy for operative management of stabbing abdominal trauma. Am. J. Surg..

[32] Leung-Tack M., Ong E.G.P., McGuirk S. (2022). Interventional radiology and open surgery: An effective partnership for solid organ trauma. J. Pediatr. Surg..

[33] Redmond E.J., Kiddoo D.A., Metcalfe P.D. (2020). Contemporary management of pediatric high grade renal trauma: 10 year experience at a level 1 trauma centre. J. Pediatr. Urol..

[34] Mahran A., Fernstrum A., Swindle M., Mishra K., Bukavina L., Raina R., Narayanamurthy V., Ross J., Woo L. (2020). Impact of trauma center designation in pediatric renal trauma: National Trauma Data Bank analysis. J. Pediatr. Urol..

[35] Jung H.S., Jeon C.H., Seo S.H. (2023). Clinical Role of Interventional Radiology in Abdominal Solid Organ Trauma. J. Korean Soc. Radiol..

[36] Lombardo C., Lopez-Ben S., Boggi U., Gutowski P., Hrbac T., Krska L., Marquez-Rivas J., Russello D., York E., Zacharias M. (2022). Hemopatch^®^ is effective and safe to use: Real-world data from a prospective European registry study. Updates Surg..

[37] Ghasemi-Rad M., Shastri R., Amaresh A., Wynne D., Whigham C. (2023). Trans-Arterial Balloon-Assisted Embolization of Traumatic Giant Hepatic Pseudoaneurysm with Thrombin. Vasc. Endovasc. Surg..

[38] Huang M., Cai S., Su J. (2019). The Pathogenesis of Sepsis and Potential Therapeutic Targets. Int. J. Mol. Sci..

[39] Strnad P., Tacke F., Koch A., Trautwein C. (2017). Liver-guardian, modifier and target of sepsis. Nat. Rev. Gastroenterol. Hepatol..

[40] Brunese L., Mercaldo F., Reginelli A., Santone A. (2020). Formal methods for prostate cancer Gleason score and treatment prediction using radiomic biomarkers. Magn. Reson. Imaging.

[41] Nainamalai V., Prasad P.J.R., Pelanis E., Edwin B., Albregtsen F., Elle O.J., Kumar R.P. (2022). Evaluation of clinical applicability of automated liver parenchyma segmentation of multi-center magnetic resonance images. Eur. J. Radiol. Open.

[42] Alirr O.I. (2020). Deep learning and level set approach for liver and tumor segmentation from CT scans. J. Appl. Clin. Med. Phys..

[43] Vorontsov E., Cerny M., Régnier P., Di Jorio L., Pal C.J., Lapointe R., Vandenbroucke-Menu F., Turcotte S., Kadoury S., Tang A. (2019). Deep Learning for Automated Segmentation of Liver Lesions at CT in Patients with Colorectal Cancer Liver Metastases. Radiol. Artif. Intell..

[44] Brunese M.C., Fantozzi M.R., Fusco R., De Muzio F., Gabelloni M., Danti G., Borgheresi A., Palumbo P., Bruno F., Gandolfo N. (2023). Update on the Applications of Radiomics in Diagnosis, Staging, and Recurrence of Intrahepatic Cholangiocarcinoma. Diagnostics.

[45] Rocca A., Brunese M.C., Santone A., Avella P., Bianco P., Scacchi A., Scaglione M., Bellifemine F., Danzi R., Varriano G. (2021). Early Diagnosis of Liver Metastases from Colorectal Cancer through CT Radiomics and Formal Methods: A Pilot Study. J. Clin. Med..

[46] Reginelli A., Vanzulli A., Sgrazzutti C., Caschera L., Serra N., Raucci A., Urraro F., Cappabianca S. (2017). Vascular microinvasion from hepatocellular carcinoma: CT findings and pathologic correlation for the best therapeutic strategies. Med. Oncol..

[47] Grassi R., Belfiore M.P., Montanelli A., Patelli G., Urraro F., Giacobbe G., Fusco R., Granata V., Petrillo A., Sacco P. (2021). COVID-19 pneumonia: Computer-aided quantification of healthy lung parenchyma, emphysema, ground glass and consolidation on chest computed tomography (CT). Radiol. Med..

[48] Kapetanou E., Malamas S., Leventis D., Karantanas A.H., Klontzas M.E. (2024). Developing a Radiomics Atlas Dataset of normal Abdominal and Pelvic computed Tomography (RADAPT). J. Imaging Inf. Med..

[49] Yamada A., Kamagata K., Hirata K., Ito R., Nakaura T., Ueda D., Fujita S., Fushimi Y., Fujima N., Matsui Y. (2023). Clinical applications of artificial intelligence in liver imaging. Radiol. Med..

[50] Schlanger D., Graur F., Popa C., Moiș E., Al Hajjar N. (2022). The role of artificial intelligence in pancreatic surgery: A systematic review. Updates Surg..

[51] Boggi U. (2023). Precision surgery. Updates Surg..

[52] Granata V., Fusco R., De Muzio F., Cutolo C., Setola S.V., Grassi R., Grassi F., Ottaiano A., Nasti G., Tatangelo F. (2022). Radiomics textural features by MR imaging to assess clinical outcomes following liver resection in colorectal liver metastases. Radiol. Med..

[53] Brunese L., Brunese M.C., Carbone M., Ciccone V., Mercaldo F., Santone A. (2022). Automatic PI-RADS assignment by means of formal methods. Radiol. Med..

[54] Granata V., Fusco R., Setola S.V., Galdiero R., Maggialetti N., Silvestro L., De Bellis M., Di Girolamo E., Grazzini G., Chiti G. (2023). Risk Assessment and Pancreatic Cancer: Diagnostic Management and Artificial Intelligence. Cancers.

[55] Laino M.E., Fiz F., Morandini P., Costa G., Maffia F., Giuffrida M., Pecorella I., Gionso M., Wheeler D.R., Cambiaghi M. (2023). A virtual biopsy of liver parenchyma to predict the outcome of liver resection. Updates Surg..

[56] Newhook T.E., Tsai S., Meric-Bernstam F. (2024). Precision Oncology in Hepatopancreatobiliary Cancer Surgery. Surg. Oncol. Clin. N. Am..

[57] Brunese L., Martinelli F., Mercaldo F., Santone A. (2020). Machine learning for coronavirus COVID-19 detection from chest X-rays. Procedia Comput. Sci..

[58] Mori M., Palumbo D., De Cobelli F., Fiorino C. (2023). Does radiomics play a role in the diagnosis, staging and re-staging of gastroesophageal junction adenocarcinoma?. Updates Surg..

[59] Viganò L., Ammirabile A., Zwanenburg A. (2023). Radiomics in liver surgery: Defining the path toward clinical application. Updates Surg..

[60] Durso A.M., Paes F.M., Caban K., Danton G., Braga T.A., Sanchez A., Munera F. (2020). Evaluation of penetrating abdominal and pelvic trauma. Eur. J. Radiol..

[61] Zeng Y., Yang F., Hu X., Zhu F., Chen W., Lin W. (2023). Radiological predictive factors of transmural intestinal necrosis in acute mesenteric ischemia: Systematic review and meta-analysis. Eur. Radiol..

[62] Charalambous S., Klontzas M.E., Kontopodis N., Ioannou C.V., Perisinakis K., Maris T.G., Damilakis J., Karantanas A., Tsetis D. (2022). Radiomics and machine learning to predict aggressive type 2 endoleaks after endovascular aneurysm repair: A proof of concept. Acta Radiol..

[63] Rezaeitaleshmahalleh M., Mu N., Lyu Z., Zhou W., Zhang X., Rasmussen T.E., McBane R.D., Jiang J. (2023). Radiomic-based Textural Analysis of Intraluminal Thrombus in Aortic Abdominal Aneurysms: A Demonstration of Automated Workflow. J. Cardiovasc. Transl. Res..

[64] Farzaneh N., Stein E.B., Soroushmehr R., Gryak J., Najarian K. (2022). A deep learning framework for automated detection and quantitative assessment of liver trauma. BMC Med. Imaging.

[65] Huang S., Zhou Z., Qian X., Li D., Guo W., Dai Y. (2022). Automated quantitative assessment of pediatric blunt hepatic trauma by deep learning-based CT volumetry. Eur. J. Med. Res..

[66] Dreizin D., Chen T., Liang Y., Zhou Y., Paes F., Wang Y., Yuille A.L., Roth P., Champ K., Li G. (2021). Added value of deep learning-based liver parenchymal CT volumetry for predicting major arterial injury after blunt hepatic trauma: A decision tree analysis. Abdom. Radiol..

[67] Levy B.E., Castle J.T., Virodov A., Wilt W.S., Bumgardner C., Brim T., McAtee E., Schellenberg M., Inaba K., Warriner Z. (2023). Artificial Intelligence Evaluation of Focused Assessment with Sonography in Trauma. J. Trauma Acute Care Surg..

[68] Wang H., Bao Q., Cao D., Dong S., Wu L. (2022). Characteristics of Computed Tomography Images for Patients with Acute Liver Injury Caused by Sepsis under Deep Learning Algorithm. Contrast Media Mol. Imaging.

[69] Alessandrino F., Qin L., Cruz G., Sahu S., Rosenthal M.H., Meyerhardt J.A., Shinagare A.B. (2019). 5-Fluorouracil induced liver toxicity in patients with colorectal cancer: Role of computed tomography texture analysis as a potential biomarker. Abdom. Radiol..

[70] Tinkoff G., Esposito T.J., Reed J., Kilgo P., Fildes J., Pasquale M., Meredith J.W. (2008). American Association for the Surgery of Trauma Organ Injury Scale I: Spleen, liver, and kidney, validation based on the National Trauma Data Bank. J. Am. Coll. Surg..

[71] Kobashi H., Toshimori J., Yamamoto K. (2013). Sepsis-associated liver injury: Incidence, classification and the clinical significance. Hepatol. Res..

[72] Dong V., Nanchal R., Karvellas C.J. (2020). Pathophysiology of Acute Liver Failure. Nutr. Clin. Pract..

[73] Lelubre C., Vincent J.L. (2018). Mechanisms and treatment of organ failure in sepsis. Nat. Rev. Nephrol..

[74] Woźnica E.A., Inglot M., Woźnica R.K., Łysenko L. (2018). Liver dysfunction in sepsis. Adv. Clin. Exp. Med..

[75] Vall A., Sabnis Y., Shi J., Class R., Hochreiter S., Klambauer G. (2021). The Promise of AI for DILI Prediction. Front. Artif. Intell..

[76] Williams D.P., Lazic S.E., Foster A.J., Semenova E., Morgan P. (2020). Predicting Drug-Induced Liver Injury with Bayesian Machine Learning. Chem. Res. Toxicol..

[77] Minerali E., Foil D.H., Zorn K.M., Lane T.R., Ekins S. (2020). Comparing Machine Learning Algorithms for Predicting Drug-Induced Liver Injury (DILI). Mol. Pharm..

[78] Costa G., Cavinato L., Masci C., Fiz F., Sollini M., Politi L.S., Chiti A., Balzarini L., Aghemo A., di Tommaso L. (2021). Virtual Biopsy for Diagnosis of Chemotherapy-Associated Liver Injuries and Steatohepatitis: A Combined Radiomic and Clinical Model in Patients with Colorectal Liver Metastases. Cancers.

[79] Asai Y., Ooi H., Sato Y. (2023). Risk evaluation of carbapenem-induced liver injury based on machine learning analysis. J. Infect. Chemother..

[80] Lai N.H., Shen W.C., Lee C.N., Chang J.C., Hsu M.C., Kuo L.N., Yu M.C., Chen H.Y. (2020). Comparison of the predictive outcomes for anti-tuberculosis drug-induced hepatotoxicity by different machine learning techniques. Comput. Methods Programs Biomed..

[81] Puri M. (2020). Automated Machine Learning Diagnostic Support System as a Computational Biomarker for Detecting Drug-Induced Liver Injury Patterns in Whole Slide Liver Pathology Images. Assay Drug Dev. Technol..

[82] Wong Y.C., Wang L.J., Wu C.H., Chen H.W., Yuan K.C., Hsu Y.P., Lin B.C., Kang S.C. (2020). Differences of liver CT perfusion of blunt trauma treated with therapeutic embolization and observation management. Sci. Rep..

[83] Yoon W., Jeong Y.Y., Kim J.K., Seo J.J., Lim H.S., Shin S.S., Kim J.C., Jeong S.W., Park J.G., Kang H.K. (2005). CT in blunt liver trauma. Radiographics.

[84] Goodman D.A., Tiruchelvam V., Tabb D.R., Agarwal N., Rhoads J.E. (1995). 3D CT reconstruction in the surgical management of hepatic injuries. Ann. R. Coll. Surg. Engl..

[85] Kozar R.A., Crandall M., Shanmuganathan K., Zarzaur B.L., Coburn M., Cribari C., Kaups K., Schuster K., Tominaga G.T. (2018). Organ injury scaling 2018 update: Spleen, liver, and kidney. J. Trauma Acute Care Surg..

[86] Homann G., Toschke C., Gassmann P., Vieth V. (2014). Accuracy of the AAST organ injury scale for CT evaluation of traumatic liver and spleen injuries. Chin. J. Traumatol..

[87] Morell-Hofert D., Primavesi F., Fodor M., Gassner E., Kranebitter V., Braunwarth E., Haselbacher M., Nitsche U.P., Schmid S., Blauth M. (2020). Validation of the revised 2018 AAST-OIS classification and the CT severity index for prediction of operative management and survival in patients with blunt spleen and liver injuries. Eur. Radiol..

[88] Paydar S., Mahmoodi M., Jamshidi M., Niakan H., Keshavarz M., Moeenvaziri N., Ghorbaninejad M.E., Farrokhnia F., Izadi Fard F., Jaafari Z. (2014). Perihepatic Packing versus Primary Surgical Repair in Patients with Blunt Liver Trauma; an 8-year Experience. Bull. Emerg. Trauma.

[89] Schullian P., Weiss H., Klaus A., Widmann G., Kranewitter C., Mittermair C., Margreiter R., Bale R. (2014). Laparoscopic liver packing to protect surrounding organs during thermal ablation. Minim. Invasive Ther. Allied Technol..

[90] Küçükaslan H., Tayar S., Oğuz Ş., Topaloglu S., Geze Saatci S., Şenel A.C., Calik A. (2022). The role of liver resection in the management of severe blunt liver trauma. Ulus. Travma Acil Cerrahi Derg..

[91] Kaptanoglu L., Kurt N., Sikar H.E. (2017). Current approach to liver traumas. Int. J. Surg..

[92] Reginelli A., Mandato Y., Solazzo A., Berritto D., Iacobellis F., Grassi R. (2012). Errors in the radiological evaluation of the alimentary tract: Part II. Semin. Ultrasound CT MRI.

[93] Pang Q., Zhou S., Liu S., Liu H., Lu Z. (2022). Prognostic role of preoperative albumin-bilirubin score in posthepatectomy liver failure and mortality: A systematic review and meta-analysis. Updates Surg..

[94] Gerardo R.G., Ponsky T.A. (2021). Diagnostic Laparoscopy for Abdominal Trauma in Infants and Children: How We Do It. J. Laparoendosc. Adv. Surg. Tech. A.

[95] Ceccarelli G., Pasculli A., Bugiantella W., De Rosa M., Catena F., Rondelli F., Costa G., Rocca A., Longaroni M., Testini M. (2020). Minimally invasive laparoscopic and robot-assisted emergency treatment of strangulated giant hiatal hernias: Report of five cases and literature review. World J. Emerg. Surg..

[96] Beltzer C., Bachmann R., Strohäker J., Axt S., Schmidt R., Küper M., Königsrainer A. (2020). Value of laparoscopy in blunt and penetrating abdominal trauma—A systematic review. Chirurg.

[97] Green M.H., Duell R.M., Johnson C.D., Jamieson N.V. (2001). Haemobilia. Br. J. Surg..

[98] Murugesan S.D., Sathyanesan J., Lakshmanan A., Ramaswami S., Perumal S., Perumal S.U., Ramasamy R., Palaniappan R. (2014). Massive hemobilia: A diagnostic and therapeutic challenge. World J. Surg..

[99] Wang H.-W., Jin K.-M., Li J., Wang K., Xing B.-C. (2022). Postoperative complications predict poor outcomes only in patients with a low modified clinical score after resection of colorectal liver metastases: A retrospective cohort study. Updat. Surg..

[100] Amato B., Compagna R., Rocca A., Bianco T., Milone M., Sivero L., Vigliotti G., Amato M., Danzi M., Aprea G. (2016). Fondaparinux vs warfarin for the treatment of unsuspected pulmonary embolism in cancer patients. Drug Des. Dev. Ther..

[101] Cazauran J.B., Muller A., Hengy B., Valette P.J., Gruner L., Monneuse O. (2018). Preliminary Report of Percutaneous Cholecystostomy as Diagnosis and Treatment of Biliary Tract Trauma. World J. Surg..

[102] Calamia S., Barbara M., Cipolla C., Grassi N., Pantuso G., Li Petri S., Pagano D., Gruttadauria S. (2022). Risk factors for bile leakage after liver resection for neoplastic disease. Updates Surg..

[103] Gachabayov M., Kubachev K., Mityushin S., Zarkua N. (2017). Recurrent Hemobilia Due to Right Hepatic Artery Pseudoaneurysm. Clin. Med. Res..

[104] Nicol A.J., Hommes M., Primrose R., Navsaria P.H., Krige J.E. (2007). Packing for control of hemorrhage in major liver trauma. World J. Surg..

[105] Perrella A., Giuliani A., De Palma M., Castriconi M., Molino C., Vennarecci G., Antropoli C., Esposito C., Calise F., Frangiosa A. (2022). C-reactive protein but not procalcitonin may predict antibiotic response and outcome in infections following major abdominal surgery. Updates Surg..

[106] Guglielmo N., Melandro F., Improta L., Mennini G., Rossi D., Assenza M., Rossi M., Berloco P.B. (2015). Early right hepatectomy for severe liver trauma: A case report. Clin. Ter..

[107] Chen Y.L., Kuo S.J., Yang L.H., Chen S.T., Tsai J.H., Chang H.C. (2003). Extrahepatic division of the right hepatic vein in right hepatectomy for blunt liver trauma. Int. Surg..

[108] Guglielmi A., Tripepi M., Salmaso L., Fedeli U., Ruzzenente A., Saia M. (2023). Trends in hospital volume and operative mortality in hepato-biliary surgery in Veneto region, Italy. Updates Surg..

[109] Calise F., Giuliani A., Sodano L., Crolla E., Bianco P., Rocca A., Ceriello A. (2015). Segmentectomy: Is minimally invasive surgery going to change a liver dogma?. Updates Surg..

[110] Berardi G., Guglielmo N., Colasanti M., Meniconi R.L., Ferretti S., Mariano G., Usai S., Angrisani M., Pecoraro A., Lucarini A. (2022). Associating liver partition and portal vein ligation for staged hepatectomy (ALPPS) for advanced hepatocellular carcinoma with macrovascular invasion. Updates Surg..

[111] Carver D., Kirkpatrick A.W., D’Amours S., Hameed S.M., Beveridge J., Ball C.G. (2020). A Prospective Evaluation of the Utility of a Hybrid Operating Suite for Severely Injured Patients: Overstated or Underutilized?. Ann. Surg..

[112] Di Benedetto F., Magistri P., Guerrini G.P., Di Sandro S. (2022). Robotic liver partition and portal vein embolization for staged hepatectomy for perihilar cholangiocarcinoma. Updates Surg..

[113] Pillai A.S., Kumar G., Pillai A.K. (2021). Hepatic Trauma Interventions. Semin. Interv. Radiol..

[114] Avella P., Vaschetti R., Cappuccio M., Gambale F., DE Meis L., Rafanelli F., Brunese M.C., Guerra G., Scacchi A., Rocca A. (2022). The role of liver surgery in simultaneous synchronous colorectal liver metastases and colorectal cancer resections: A literature review of 1730 patients underwent open and minimally invasive surgery. Minerva Surg..

[115] Rocca A., Cipriani F., Belli G., Berti S., Boggi U., Bottino V., Cillo U., Cescon M., Cimino M., Corcione F. (2021). The Italian Consensus on minimally invasive simultaneous resections for synchronous liver metastasis and primary colorectal cancer: A Delphi methodology. Updates Surg..

[116] Chiow A.K., Lee S.Y., Chan C.Y., Tan S.S. (2015). Learning curve in laparoscopic liver surgery: A fellow’s perspective. Hepatobiliary Surg. Nutr..

[117] Mascarenhas A., Marques H.P., Coutinho J., Martins A., Nolasco F. (2023). Liver cirrhosis requiring transplantation in the context of hepaticojejunostomy stricture after a traumatic bile duct injury. Radiol. Case Rep..

[118] Dogeas E., Tohme S., Geller D.A. (2021). Laparoscopic liver resection: Global diffusion and learning curve. Ann. Acad. Med. Singap..

[119] Ceccarelli G., Valeri M., Amato L., De Rosa M., Rondelli F., Cappuccio M., Gambale F.E., Fantozzi M., Sciaudone G., Avella P. (2023). Robotic revision surgery after failed Nissen anti-reflux surgery: A single center experience and a literature review. J. Robot. Surg..

[120] Nti B.K., Laniewicz M., Skaggs T., Cross K., Fallat M.E., Rominger A. (2019). A novel streamlined trauma response team training improves imaging efficiency for pediatric blunt abdominal trauma patients. J. Pediatr. Surg..

[121] Matsevych O.Y., Koto M.Z., Aldous C. (2018). Trauma laparoscopy: A prospect of skills training (cohort study). Int. J. Surg..

[122] Buondonno A., Avella P., Cappuccio M., Scacchi A., Vaschetti R., Di Marzo G., Maida P., Luciani C., Amato B., Brunese M.C. (2022). A Hub and Spoke Learning Program in Bariatric Surgery in a Small Region of Italy. Front. Surg..

[123] Saviano A., Ojetti V., Zanza C., Franceschi F., Longhitano Y., Martuscelli E., Maiese A., Volonnino G., Bertozzi G., Ferrara M. (2022). Liver Trauma: Management in the Emergency Setting and Medico-Legal Implications. Diagnostics.

[124] Giuliani A., Avella P., Segreto A.L., Izzo M.L., Buondonno A., Coluzzi M., Cappuccio M., Brunese M.C., Vaschetti R., Scacchi A. (2021). Postoperative Outcomes Analysis after Pancreatic Duct Occlusion: A Safe Option to Treat the Pancreatic Stump After Pancreaticoduodenectomy in Low-Volume Centers. Front. Surg..

[125] Kosola J., Brinck T., Leppäniemi A., Handolin L. (2020). Blunt Abdominal Trauma in a European Trauma Setting: Need for Complex or Non-Complex Skills in Emergency Laparotomy. Scand. J. Surg..

[126] Peris A., Mangini M., Spina R., Franci A., Ognibene A., Zagli G. (2013). Application of emergency trauma score in a hub-and-spoke regional system. Br. J. Anaesth..

[127] Pang C., Chen Z.D., Wei B., Xu W.T., Xi H.Q. (2022). Military training-related abdominal injuries and diseases: Common types, prevention and treatment. Chin. J. Traumatol..

[128] Raum M.R., Nijsten M.W., Vogelzang M., Schuring F., Lefering R., Bouillon B., Rixen D., Neugebauer E.A., Ten Duis H.J., Polytrauma Study Group of the German Trauma Society (2009). Emergency trauma score: An instrument for early estimation of trauma severity. Crit. Care Med..

[129] Bhan C., Forshaw M.J., Bew D.P., Kapadia Y.K. (2007). Diagnostic peritoneal lavage and ultrasonography for blunt abdominal trauma: Attitudes and training of current general surgical trainees. Eur. J. Emerg. Med..

[130] Loffredo D., Marvaso A., Ceraso S., Cinelli N., Rocca A., Vitale M., Rossi M., Genovese E., Amato B., Cinelli M. (2013). Minimal invasive surgery in treatment of liver metastases from colorectal carcinomas: Case studies and survival rates. BMC Surg..

[131] Aprea G., De Rosa D., Milone M., Rocca A., Bianco T., Massa G., Compagna R., Johnson L.B., Sanguinetti A., Polistena A. (2017). Laparoscopic distal pancreatectomy in elderly patients: Is it safe?. Aging Clin. Exp. Res..

[132] Rocca A., Scacchi A., Cappuccio M., Avella P., Bugiantella W., De Rosa M., Costa G., Polistena A., Codacci-Pisanelli M., Amato B. (2021). Robotic surgery for colorectal liver metastases resection: A systematic review. Int. J. Med. Robot..

[133] Justin V., Fingerhut A., Uranues S. (2017). Laparoscopy in Blunt Abdominal Trauma: For Whom? When?and Why?. Curr. Trauma Rep..

[134] Pau L., Navez J., Cawich S.O., Dapri G. (2021). Laparoscopic Management of Blunt and Penetrating Abdominal Trauma: A Single-Center Experience and Review of the Literature. J. Laparoendosc. Adv. Surg. Tech. A.

[135] Famularo S., Berardi G., Pawlik T.M., Donadon M., Torzilli G. (2022). Liver drains after surgery: What is the real practice? An international snapshot from the Li.DR.A.S. survey. Updates Surg..

[136] Aboud E.T., Krisht A.F., O’Keeffe T., Nader R., Hassan M., Stevens C.M., Ali F., Luchette F.A. (2011). Novel simulation for training trauma surgeons. J. Trauma.

[137] Aldrighetti L., Boggi U., Falconi M., Giuliante F., Cipriani F., Ratti F., Torzilli G. (2020). Perspectives from Italy during the COVID-19 pandemic: Nationwide survey-based focus on minimally invasive HPB surgery. Updates Surg..

[138] Ceccarelli G., Rocca A., De Rosa M., Fontani A., Ermili F., Andolfi E., Bugiantella W., Levi Sandri G.B. (2021). Minimally invasive robotic-assisted combined colorectal and liver excision surgery: Feasibility, safety and surgical technique in a pilot series. Updates Surg..

[139] Kaur S., Bagaria D., Kumar A., Priyadarshini P., Choudhary N., Sagar S., Gupta A., Mishra B., Joshi M., Kumar A. (2023). Contrast-enhanced computed tomography abdomen versus diagnostic laparoscopy-based management in patients with penetrating abdominal trauma: A randomised controlled trial. Eur. J. Trauma Emerg. Surg..

[140] Ceccarelli G., Costa G., De Rosa M., Codacci Pisanelli M., Frezza B., De Prizio M., Bravi I., Scacchi A., Gallo G., Amato B. (2021). Minimally Invasive Approach to Gastric GISTs: Analysis of a Multicenter Robotic and Laparoscopic Experience with Literature Review. Cancers.

[141] Marks J.M., Youngelman D.F., Berk T. (1997). Cost analysis of diagnostic laparoscopy vs laparotomy in the evaluation of penetrating abdominal trauma. Surg. Endosc..

[142] Katlic M.R. (2021). Let It Rain: The American College of Surgeons Geriatric Surgery Verification Program. J. Am. Geriatr. Soc..

[143] Rocca A., Brunese M.C., Cappuccio M., Scacchi A., Martucci G., Buondonno A., Perrotta F.M., Quarto G., Avella P., Amato B. (2021). Impact of Physical Activity on Disability Risk in Elderly Patients Hospitalized for Mild Acute Diverticulitis and Diverticular Bleeding Undergone Conservative Management. Medicina.

[144] Zha J., Ni J., Chen S., Feng H., Che T., Qiao S. (2022). Ultrasound Radiomics-Guided Iliac Fascia Block on Postoperative Cognitive Dysfunction in Elderly Patients Undergoing Hip Surgery. Comput. Math. Methods Med..

[145] Guerra G., Testa D., Montagnani S., Tafuri D., Salzano F.A., Rocca A., Amato B., Salzano G., Dell’Aversana Orabona G., Piombino P. (2014). Surgical management of pleomorphic adenoma of parotid gland in elderly patients: Role of morphological features. Int. J. Surg..

[146] Komici K., Dello Iacono A., De Luca A., Perrotta F., Bencivenga L., Rengo G., Rocca A., Guerra G. (2021). Adiponectin and Sarcopenia: A Systematic Review with Meta-Analysis. Front. Endocrinol..

[147] Aprea G., Rocca A., Salzano A., Sivero L., Scarpaleggia M., Ocelli P., Amato M., Bianco T., Serra R., Amato B. (2016). Laparoscopic single site (LESS) and classic video-laparoscopic cholecystectomy in the elderly: A single centre experience. Int. J. Surg..

[148] Tamura K., Nakamori M., Matsuda K., Hotta T., Nakamura M., Yokoyama S., Iwahashi M., Yamade N., Yamaue H. (2023). Elective colorectal cancer surgery in nonagenarians and postoperative outcomes. Updates Surg..

[149] Rocca A., Porfidia C., Russo R., Tamburrino A., Avella P., Vaschetti R., Bianco P., Calise F. (2023). Neuraxial anesthesia in hepato-pancreatic-bilio surgery: A first western pilot study of 46 patients. Updates Surg..

[150] Komici K., Cappuccio M., Scacchi A., Vaschetti R., Delli Carpini G., Picerno V., Avella P., Brunese M.C., Rengo G., Guerra G. (2022). The Prevalence and the Impact of Frailty in Hepato-Biliary Pancreatic Cancers: A Systematic Review and Meta-Analysis. J. Clin. Med..

[151] Luciani C., Scacchi A., Vaschetti R., Di Marzo G., Fatica I., Cappuccio M., Guerra G., Ceccarelli G., Avella P., Rocca A. (2022). The uniportal VATS in the treatment of stage II pleural empyema: A safe and effective approach for adults and elderly patients-a single-center experience and literature review. World J. Emerg. Surg..

[152] Triantafyllou E., Woollard K.J., McPhail M.J.W., Antoniades C.G., Possamai L.A. (2018). The Role of Monocytes and Macrophages in Acute and Acute-on-Chronic Liver Failure. Front. Immunol..

[153] Surewaard B.G.J., Thanabalasuriar A., Zeng Z., Tkaczyk C., Cohen T.S., Bardoel B.W., Jorch S.K., Deppermann C., Bubeck Wardenburg J., Davis R.P. (2018). α-Toxin Induces Platelet Aggregation and Liver Injury during Staphylococcus aureus Sepsis. Cell Host Microbe.

[154] Ndomba N., Soldera J. (2023). Management of sepsis in a cirrhotic patient admitted to the intensive care unit: A systematic literature review. World J. Hepatol..

[155] Brillantino A., Iacobellis F., Brusciano L., Abu-Omar A., Muto G., Amadu A.M., Foroni F., Antropoli M., Antropoli C., Castriconi M. (2023). Accuracy of computed tomography in staging acute appendicitis and its impact on surgical outcome and strategy: A multi-center retrospective case-control study. Radiol. Med..

[156] Granata V., Fusco R., Cozzi D., Danti G., Faggioni L., Buccicardi D., Prost R., Ferrari R., Trinci M., Galluzzo M. (2023). Structured reporting of computed tomography in the polytrauma patient assessment: A Delphi consensus proposal. Radiol. Med..

[157] Tujios S., Stravitz R.T., Lee W.M. (2022). Management of Acute Liver Failure: Update 2022. Semin. Liver Dis..

[158] Boldingh Q.J., de Vries F.E., Boermeester M.A. (2017). Abdominal sepsis. Curr. Opin. Crit. Care.

[159] Crouser E.D., Parrillo J.E., Seymour C., Angus D.C., Bicking K., Tejidor L., Magari R., Careaga D., Williams J., Closser D.R. (2017). Improved Early Detection of Sepsis in the ED with a Novel Monocyte Distribution Width Biomarker. Chest.

[160] Carrigan S.D., Scott G., Tabrizian M. (2004). Toward resolving the challenges of sepsis diagnosis. Clin. Chem..

[161] Sarin S.K., Choudhury A., Sharma M.K., Maiwall R., Al Mahtab M., Rahman S., Saigal S., Saraf N., Soin A.S., Devarbhavi H. (2019). Acute-on-chronic liver failure: Consensus recommendations of the Asian Pacific association for the study of the liver (APASL): An update. Hepatol. Int..

[162] Wang W., Xu C., Ma X., Zhang X., Xie P. (2020). Intensive Care Unit-Acquired Weakness: A Review of Recent Progress with a Look Toward the Future. Front. Med..

[163] Lee C.C., Ho C.Y., Chen P.L., Hsieh C.C., Wang W.Y.C., Lin C.H., Ko W.C. (2021). Is qSOFA Suitable for Early Diagnosis of Sepsis Among Bacteremia Patients in Emergency Departments? Time for a Reappraisal of Sepsis-3 Criteria. Front. Med..

